# Accumulation of exhausted CD8+ T cells in extramammary Paget’s disease

**DOI:** 10.1371/journal.pone.0211135

**Published:** 2019-01-25

**Authors:** Natsuko Iga, Atsushi Otsuka, Yosuke Yamamoto, Chisa Nakashima, Tetsuya Honda, Akihiko Kitoh, Saeko Nakajima, Gyohei Egawa, Takashi Nomura, Teruki Dainichi, Shigeto Matsushita, Hideaki Tanizaki, Yuki Yamamoto, Takeru Funakoshi, Yasuhiro Fujisawa, Taku Fujimura, Hiroo Hata, Yoshihiro Ishida, Kenji Kabashima

**Affiliations:** 1 Department of Dermatology, Kyoto University Graduate School of Medicine, Kyoto, Japan; 2 Translational Research Department for Skin and Brain Diseases, Kyoto University Graduate School of Medicine, Kyoto, Japan; 3 Department of Dermato-Oncology/Dermatology, National Hospital Organization Kagoshima Medical Center, Kagoshima, Japan; 4 Department of Dermatology, Osaka Medical College, Osaka, Japan; 5 Department of Dermatology, Wakayama Medical University, Wakayama, Japan; 6 Department of Dermatology, Keio University School of Medicine, Tokyo, Japan; 7 Department of Dermatology, University of Tsukuba, Tsukuba, Japan; 8 Department of Dermatology, Tohoku University Graduate School of Medicine, Sendai, Japan; 9 Department of Dermatology, Hokkaido University Graduate School of Medicine, Sapporo, Japan; 10 Singapore Immunology Network (SIgN) and Institute for Medical Biology, Agency for Science, Technology and Research (A*STAR), Singapore, Singapore; University of South Alabama Mitchell Cancer Institute, UNITED STATES

## Abstract

Cancer immunotherapy has highlighted the clinical relevance of enhancing anti-tumor response of CD8+ T cells in several cancer types. Little is known, however, about the involvement of the immune system in extramammary Paget’s disease (EMPD). We examined the cytotoxicity and the effector functions of CD8+ T cells using paired samples of peripheral blood and tumors by flow cytometry. Expression levels of perforin, granzyme B, IFN-g, TNF-a, and IL-2 in CD8+ tumor-infiltrating lymphocytes (TILs) were significantly lower than those in CD8+ T cells of peripheral blood. Significantly higher expression of PD-1 was found in CD8+TILs than in CD8+ T cells of peripheral blood. A high number of CD8+ cells was significantly associated with poor overall survival (OS) adjusted with age, sex, and clinical stage (hazard ratio [HR] = 5.03, *P* = 0.045, 95% confidence interval [CI] 1.03–24.4). On the other hand, the number of PD-1+ cells was not associated with OS or disease-free survival (DFS). Moreover, we found that tumor cells produced immunosuppressive molecule indoleamine 2,3-dyoxygenae (IDO). In conclusion, CD8+ TILs displayed an exhausted phenotype in EMPD. IDO expression seemed more relevant in inducing CD8 exhaustion than PD-1 upregulation or PD-L1 expression by immune cells. Restoring the effector functions of CD8+ TILs could be an effective treatment strategy for advanced EMPD.

## Introduction

Extramammary Paget’s disease (EMPD) is a rare skin cancer that occurs predominantly in areas with abundant apocrine sweat glands including the axillary, perianal and genital regions [1]. EMPD usually presents as slow-growing carcinoma *in situ* with a favorable prognosis. However, some EMPD tumors show invasive / metastatic progression and the prognosis is dismal in such cases. Five-year survival rate is 84% in patients without metastasis, whereas only 7% in patients with distant metastasis [2]. Standard therapies for advanced EMPD are lacking, and they are often refractory to systemic therapies [3].

Cancer immunotherapy has highlighted the importance of tumor immunity. The presence of tumor-infiltrating lymphocytes (TILs) is essential for anti-tumor immune response. A high number of CD8+ TILs is associated with favorable prognosis, and a high number of tumor-infiltrating regulatory T cells (Tregs) is associated with poor prognosis in several cancer types [4,5]. The capacity of TILs to act as effector cells is hindered by the tumor microenvironment. For example, programmed death-1 (PD-1) is an immuno-inhibitory receptor expressed by lymphocytes that inhibits their proliferation and effector functions after it binds with programmed death ligand-1 (PD-L1). PD-1 upregulation on CD8+ TILs is associated with exhaustion in several cancer types [6–8]. Therefore, the expression of PD-1 or PD-L1 is associated with poor prognosis in various cancer types [9,10]. Therapeutic PD-1 blockade improved overall survival (OS) by enhancing tumor immunity [11,12]. Indoleamine 2,3-dioxygenase (IDO) is a tryptophan-metabolizing enzyme that is upregulated on tumor cells and contributes to the suppression of T cell response in several cancer types [13–15]. Combination therapy with an IDO-1 inhibitor plus checkpoint inhibitors in patients with several cancer types is being tested in a clinical trial [16]. The mechanisms of immune evasion in the tumor microenvironment have been revealed in many cancer types. However, little is known about the involvement of the immune system in EMPD.

In this study, we examined the cytotoxicity, the effector functions, and PD-1 expression of CD8+ TILs in EMPD by flow cytometry. We also evaluated the association of CD8+ cells and PD-1+ cells with prognosis. In addition, we investigated the tumor-derived immunosuppressive molecule IDO by immunohistochemistry (IHC) and quantitative polymerase chain reaction analysis (qPCR).

## Materials and methods

### Research ethics

This study protocol was approved by the ethics committee of the Kyoto University Graduate School of Medicine and participating institutions. Human samples were collected according to the procedure specified in the study protocol. Written informed consent forms were obtained from all patients.

### Samples for survival analysis

We used formalin-fixed, paraffin-embedded (FFPE) tumor specimens from 54 primary EMPD patients (stage I-III) treated with curative surgery between 2003 and 2016 at six institutions in Japan: Kyoto University Hospital, National Hospital Organization Kagoshima Medical Center, Wakayama University Hospital, Keio University Hospital, Tsukuba University Hospital, and Tohoku University Hospital. Tumor staging was performed using a proposed tumor-node-metastasis (TNM) staging system for EMPD [2].

### Immunohistochemistry and immunofluorescence

Immunohistochemical studies were performed on the FFPE tumor specimens using surgical sections of EMPD which contain both tumor and adjacent normal tissue. FFPE sections were deparaffinized and then dehydrated. After antigen retrieval, endogenous peroxidase activity was blocked by incubation with 0.3% H_2_O_2_ in methanol for 30 min. Non-specific binding of IgG was blocked using normal goat serum (Vector Laboratories, Burlingame, CA). Sections were incubated with the primary antibody overnight. IHC was performed with primary antibodies against CD8 (Clone EP1150Y; Abcam, Cambridge, MA), PD-1 (Clone NAT105; Abcam), PD-L1 (Clone SP142; Spring Bioscience, Fremont, CA), and IDO (ab55305; Abcam). Next, they were incubated with biotinylated goat anti-rabbit or biotinylated goat anti-mouse secondary antibodies, followed by incubation with streptavidin-peroxidase complex solution for 30 min. Signals were generated by incubation with 3,3-diaminobenzidine. Lastly, the sections were counterstained with hematoxylin.

### Quantification of IHC variables

The number of CD8+ cells and PD-1+ cells adjacent to the tumor was counted individually. Five independent areas with the most abundant positive cells were selected with x40 objective lens. The number of positive cells was automatically counted with a custom script written in Fiji (National Institutes of Health, Bethesda, MD) [17]. The average count was used for statistical analysis. The median values of the number of CD8+ cells and PD-1+ cells were used as cutoffs to define a high infiltration group and a low infiltration group. PD-L1 expression on immune cells and tumor cells was analyzed separately following a previously described approach [18]. PD-L1+ tumor cells were morphologically distinguished from PD-L1+ immune cells. PD-L1 positivity was defined as PD-L1 expression in more than 5% of all immune cells and more than 1% of all tumor cells.

### Flow cytometric analysis

Paired fresh peripheral blood samples and tumor samples were obtained from 10 patients with EMPD treated in 2017 at Kyoto University and Osaka Medical College. The median age was 72 years (range, 65 to 87). Seven patients were male, and 3 patients were female. Six cases were *in situ*, 1 case in Stage I, 1 case in Stage II, and 2 cases in Stage IIIb. In addition, peripheral blood samples from 7 healthy controls were analyzed as controls. Fresh peripheral blood was collected in EDTA and peripheral blood mononuclear cells (PBMCs) were isolated by density gradient centrifugation using lymphocyte separation solution (Nacalai Tesque, Kyoto, Japan). Fresh tumor samples were minced and digested overnight at 37°C with the buffer consisting of collagenase Type 4 (Worthington Biochemical Corp., Freehold, NJ), 20% FBS, and 1% penicillin-streptomycin in RPMI medium, and then passed through the sterile mesh to obtain a single suspension. Fresh PBMCs were also incubated overnight with the buffer consisting of collagenase Type 4 in RPMI medium. The following fluorescent-labelled monoclonal antibodies were used for surface and intracellular staining: TCRab-FITC (clone T10B9.1A-31; BD Biosciences, San Jose, CA), TCRab-PE/Cy7 (clone IP26; eBioscience), CD45RA-APC/Cy7 (clone HI100; BioLegend, San Diego, CA), CCR7-BUV395 (clone 150503; BD Biosciences), CD4-PerCP/Cy5.5 (clone OKT4; BioLegend), CD8-PE (clone SK1; BioLegend), CD8-Pacific Blue (clone SK1; BioLegend), PD-1-Brilliant Violet 785 (clone EH12.2H7; BioLegend), interferon (IFN)-g-PE (clone 4S.B3; eBioscience), tumor necrosis factor (TNF)-a-APC (clone Mab11; eBioscience), interleukin (IL)-2-PE/Cy7 (clone MQ1-17H12; BioLegend), granzyme B-FITC (clone GB11; BD Biosciences), perforin-APC (clone dG9; BioLegend), mouse IgG1 isotype-FITC (clone MOPC-21; BioLegend), mouse IgG1 isotype-PE (clone MOPC-21; BioLegend), mouse IgG1 isotype-APC (clone P3.6.2.8.1; eBioscience), mouse IgG1 isotype-Brilliant Violet 785 (clone MOPC-21; BioLegend), rat IgG2b isotype-PE/Cy7 (clone eB149/10H5; eBioscience), and fixable Viability Dye-eFluor (FVD) 506 (eBioscience). FVD was used to irreversibly label dead cells prior to fixation and permeabilization procedures and to exclude dead cells. For the intracellular staining of IFN-g, TNF-a, and IL-2, PBMCs and tumor tissues were stimulated for 3 hours with BD Leukocyte Activation Cocktail (phorbol myristate acetate [PMA] and ionomycin along with the Golgi inhibitor, brefeldin A). To detect intracellular protein, cells were permeabilized, fixed, and stained according to the manufacturer’s instructions using a Cytofix/Cytoperm kit (BD Biosciences). Samples were acquired using LSR Fortessa (BD Biosciences) and analyzed with Flow Jo software (Tree Star, San Carlos, CA).

### Quantitative polymerase chain reaction analysis

We collected total RNA extracted from the tumor samples of 28 patients with EMPD treated in 2017 at seven institutions: Kyoto University Hospital, National Hospital Organization Kagoshima Medical Center, Wakayama University Hospital, Keio University Hospital, Tsukuba University Hospital, Tohoku University Hospital, and Hokkaido University. We collected total RNA extracted from the normal skin samples of 18 healthy individuals from Kyoto University. To examine *IDO1* transcription. cDNA was synthesized from total RNA using a PrimeScript RT reagent kit (TaKaRa Bio, Otsu, Japan). A LightCycler 480 and a LightCycler SYBR Green I Master (Roche Diagnostics Corp, Indianapolis, IN) were used according to the manufacturer’s protocol. Following primers were used in the analysis: *GAPDH* primer 1, 5’-CATGAGAAGTATGACAACAGCCT-3’; *GAPDH* primer 2, 5’-AGTCCTTCCACGATACCAAAGT-3’; *IDO1* primer 1, 5’-AGGCAACCCCCAGCTATCAGA-3’; and *IDO1* primer 2, 5’-GCATGTCCTCCACCAGCAGTC-3’. The expression of *IDO1* was normalized to that of the control gene *GAPDH*.

### Statistical analysis

Statistical comparisons were made using Wilcoxon signed-rank test, the Mann-Whitney U test, and two-way analysis of variance (ANOVA). Survival curves were generated by the Kaplan-Meier method. Overall survival (OS) was defined as the time from the initial diagnosis until death from any cause. Patients were censored at the date of last follow-up if death had not occurred. Disease-free survival (DFS) was measured from the date of the surgery to the date of documented recurrence or death from any cause. Patients with no signs of recurrence or death were censored at the time of the most recent follow-up. The log-rank test was used for the univariate analysis. Cox-proportional hazard regression models were employed to examine the relationships between the number of CD8+ cells and prognosis, with adjustment for possible confounders (age, gender, and clinical stages). Clinical stages were divided into early disease (Stage I and II) and late disease (Stage III). *P* < 0.05 was considered statistically significant. Statistical analysis was performed using R statistical software (R Foundation for Statistical Computing, Vienna, Austria).

## Results

### CD8+ TILs display a functionally exhausted phenotype

**Fig 1:**
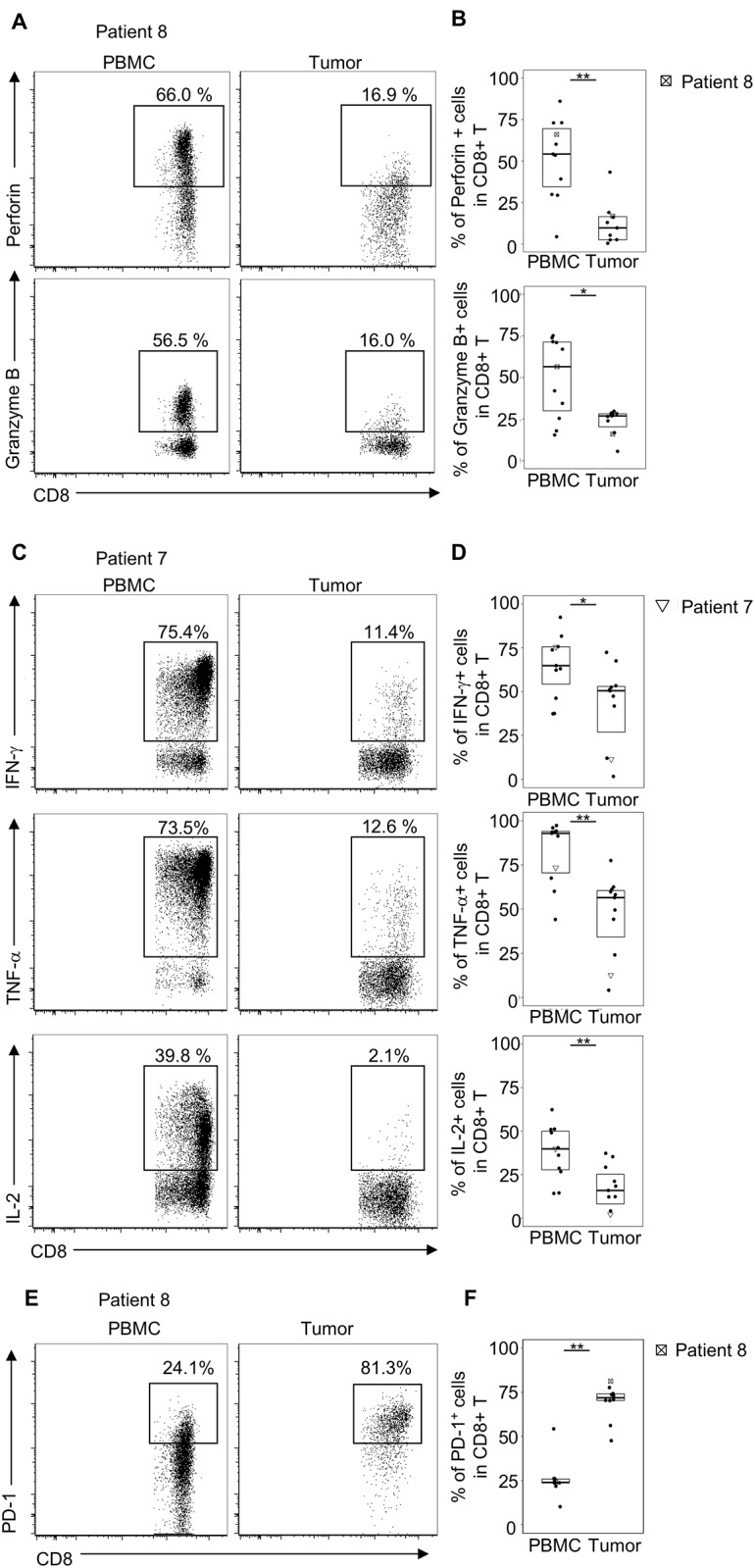
Flow cytometric analysis of perforin, granzyme B, IFN-g, TNF-a, IL-2, and PD-1 expression by CD8+ T cells in PBMCs and tumor tissues. Freshly isolated PBMCs and digested tumor tissues from 10 paired patients were stained by flow cytometry. Flow cytometric plots were pregated on TCRab+CD8+cells, excluding dead cells and naïve T cells (CD45RA+CCR7+ T cells). **(A)** Flow cytometric plots of perforin and granzyme B intracellular staining among CD8+ T cells are shown. **(B)** The frequencies of the expression levels of perforin and granzyme B in CD8+ T cells from paired PBMCs and tumor tissues are shown (n = 10). **(C)** Flow cytometric plots of IFN-g, TNF-a, and IL-2 intracellular staining among CD8+ T cells are shown. **(D)** The frequencies of the expression levels of IFN-g, TNF-a, and IL-2 in CD8+ T cells from paired PBMCs and tumor tissues are shown (n = 10). **(E)** Representative flow cytometric plots of PD-1 expression among CD8+ T cells are shown. **(F)** The frequencies of the expression levels of PD-1 in CD8+ T cells from paired PBMCs and tumor tissues are shown (n = 10). *P-*values were calculated by the Wilcoxon signed-rank test between paired PBMCs and tumor tissues. A single asterisk indicates *P* < 0.05; double asterisks indicate *P* < 0.01.

The presence of CD8+ TILs is essential for anti-tumor immune response [4,5]. To evaluate the effector functions of CD8+ TILs in EMPD patients, we analyzed the cytotoxicity and the effector functions of CD8+ T cells using paired samples of PBMCs and tumor tissues of 10 EMPD patients, following the protocol described previously [6,7,15]. We analyzed central memory T cells (CD45RA-CCR7+), effector memory T cells (CD45RA-CCR7-), and effector T cells (CD45RA+CCR7-), excluding naïve T cells (CD45RA+CCR7+) [19]. CD8+ TILs produced significantly lower levels of perforin and granzyme B (cytolytic molecules produced by cytotoxic CD8+ T cells [20]) than CD8+ T cells in PBMCs (perforin, *P* < 0.01; granzyme B, *P* < 0.05) (Fig 1A and 1B). These results demonstrated that CD8+ TILs had impaired cytotoxic activity. Next, to evaluate the effector functions of CD8+ TILs, we analyzed the cytokine expression in CD8+ T cells using paired samples of PBMCs and tumor tissues. It is well known that a loss of effector functions occurs in a stepwise manner during T cell exhaustion [21]. IL-2 production is one of the first effector activities to be extinguished, followed by the loss of TNF-a production and then IFN-g [21]. CD8+ TILs produced significantly lower levels of IFN-g, TNF-a, and IL-2 than CD8+ T cells in PBMCs (IFN-g, *P* < 0.05; TNF-a, *P* < 0.01; IL-2, *P* < 0.01) (Fig 1C and 1D). Since PD-1 upregulation on CD8+ TILs contribute to the exhaustion in several cancer types [6–8], we evaluated PD-1 expression on CD8+ T cells between 10 paired samples of PBMCs and tumor tissues by flow cytometry. The frequencies of PD-1+ T cells among CD8+ T cells were significantly higher in tumor tissues than those in PBMCs (*P* < 0.01) (Fig 1E and 1F). These results suggest that CD8+ TILs displayed a functionally exhausted phenotype.

Next, the effector functions of CD8+ T cells in both PBMCs and tumors were compared among the various T cell subsets. We found that there were differences in the effector functions of CD8+ T cells by the various T cell subsets (S1 Fig). We also compared the expression levels of perforin, granzyme B, IFN-g, TNF-a, and IL-2 of CD8+ T cells between PBMCs and tumor in each T cell subset. Almost all TILs regardless of T cell subset reduced in their functional ability, although the degree of exhaustion was variable (S1 Fig). Next, we asked if naïve CD8+ T cells in the circulation were activated in EMPD patients as compared to healthy controls. The effector functions of naïve CD8+ T cells in PBMCs were comparable between them except for TNF-a expression (S2 Fig), which suggests that there was little potential baseline activation of circulatory naïve CD8+ T cells in EMPD patients. Additionally, we compared the effector functions of CD8+ T cells in PBMCs from EMPD patients and those from healthy controls, excluding naïve T cells. All the effector functions in PBMCs were comparable except IFN-g production between them. The expression level of IFN-g was more pronouncedly downregulated in PBMCs from EMPD patients than healthy controls (S3 Fig), which indicates that CD8+ TILs might have already lost effector functions when they entered the tumor.

### A high number of CD8+ cells was associated with poor prognosis

**Fig 2:**
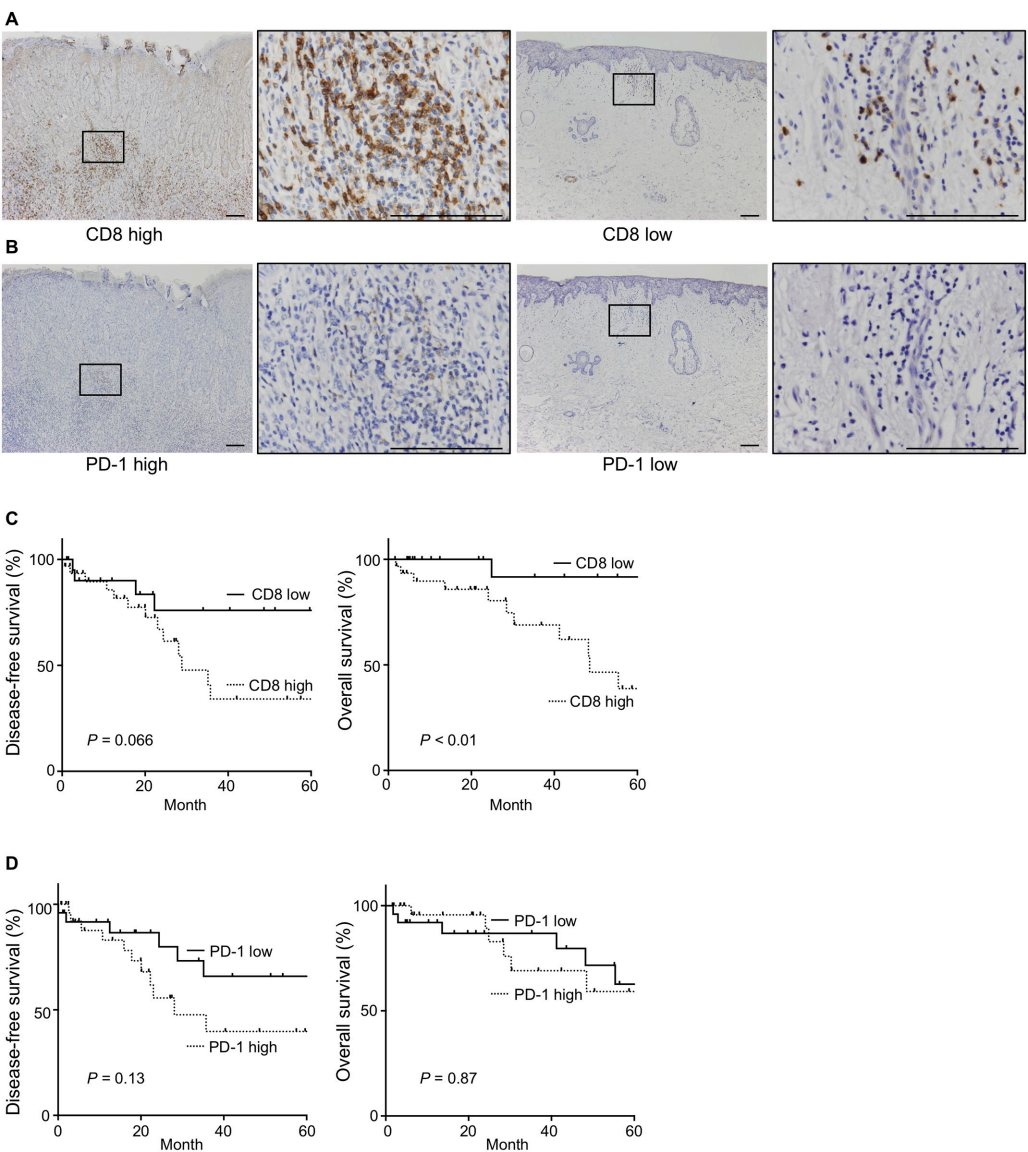
Immunohistochemical staining for CD8 and PD-1 in tumor specimens and Kaplan-Meier curves of DFS and OS stratified by the number CD8+ cells and PD-1+ cells. **(A)** Representative IHC stainings of tumor specimens with a high number of CD8+ cells and a low number of CD8+ cells are shown. **(B)** Representative IHC stainings of tumor specimens with a high number of PD-1+ cells and a low number of PD-1+ cells are shown. Scale bar, 100 μm. **(C)** Kaplan-Meier DFS and OS curves stratified by the number of CD8+ cells adjacent to the tumor (n = 54) are shown. **(D)** Kaplan-Meier DFS and OS curves stratified by the number of PD-1+ cells adjacent to the tumor (n = 54) are shown. The log-rank test was performed for the univariate analysis.

**Fig 3:**
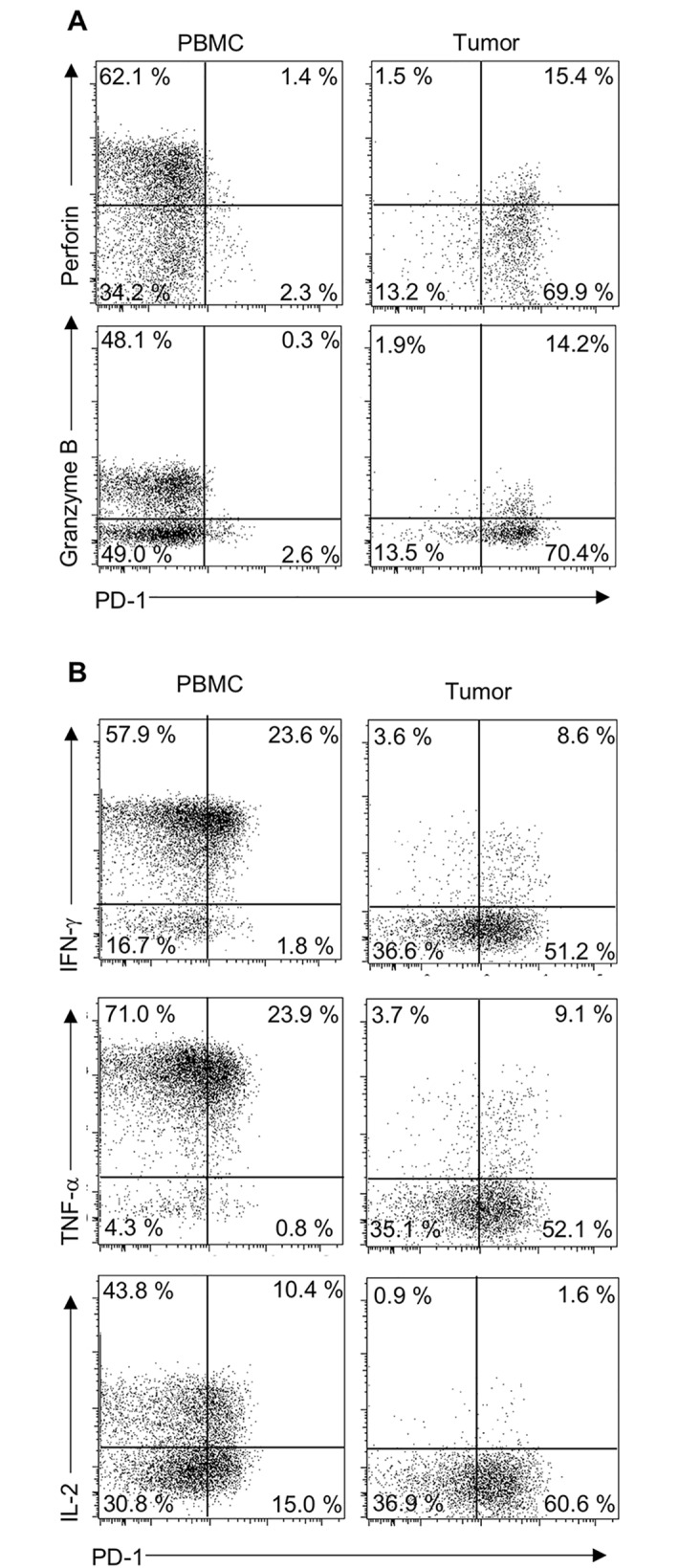
Flow cytometric analysis of PD-1 expression and the production of perforin, granzyme B, IFN-g, TNF-a, and IL-2 in CD8+ T cells in PBMCs and tumor tissues from EMPD patients. Freshly isolated PBMCs and digested tumor tissues were analyzed by flow cytometry. Flow cytometric plots were pregated on TCRab+CD8+ cells, excluding dead cells and naïve T cells (CD45RA+CCR7+ T cells). **(A)** Representative flow cytometric plots of PD-1 expression and perforin and granzyme B intracellular staining among CD8+ T cells are shown. **(B)** Representative flow cytometric plots of PD-1 expression and IFN-g, TNF-a, and IL-2 intracellular staining among CD8+ T cells are shown.

In EMPD, CD8+ TILs displayed an exhausted phenotype and PD-1 was upregulated on CD8+ TILs. Next, we examined the relationships between the number of CD8+ cells and PD-1+ cells adjacent to the tumor cells, and prognosis of 54 EMPD patients by IHC analysis (S1 Table) (Fig 2A and 2B). We found that a high number of CD8+ cells was significantly associated with poor OS (*P* < 0.01, by log rank test) (Fig 2C). Multivariate Cox-proportional hazard regression analysis revealed that a high number of CD8+ cells was significantly associated with poor OS adjusted for age, sex, and clinical stage (hazard ratio [HR] = 5.03, *P* = 0.045, 95% confidence interval [CI] 1.03–24.4) (S2 Table). On the other hand, the number of PD-1+ cells was not associated with OS or DFS (*P* = 0.87, by log rank test) (*P* = 0.13, by log rank test) (Fig 2D). In addition, the flow cytometric analysis showed that there was no correlation between the level of PD-1 expression on the CD8+ T cells and their functional ability (Fig 3A and 3B). We confirmed that most of PD-1+ cells were T cells at the tumor site by flow cytometry (mean 83.5%, range: 83.3–92.3%) (S4 Fig).

**Fig 4:**
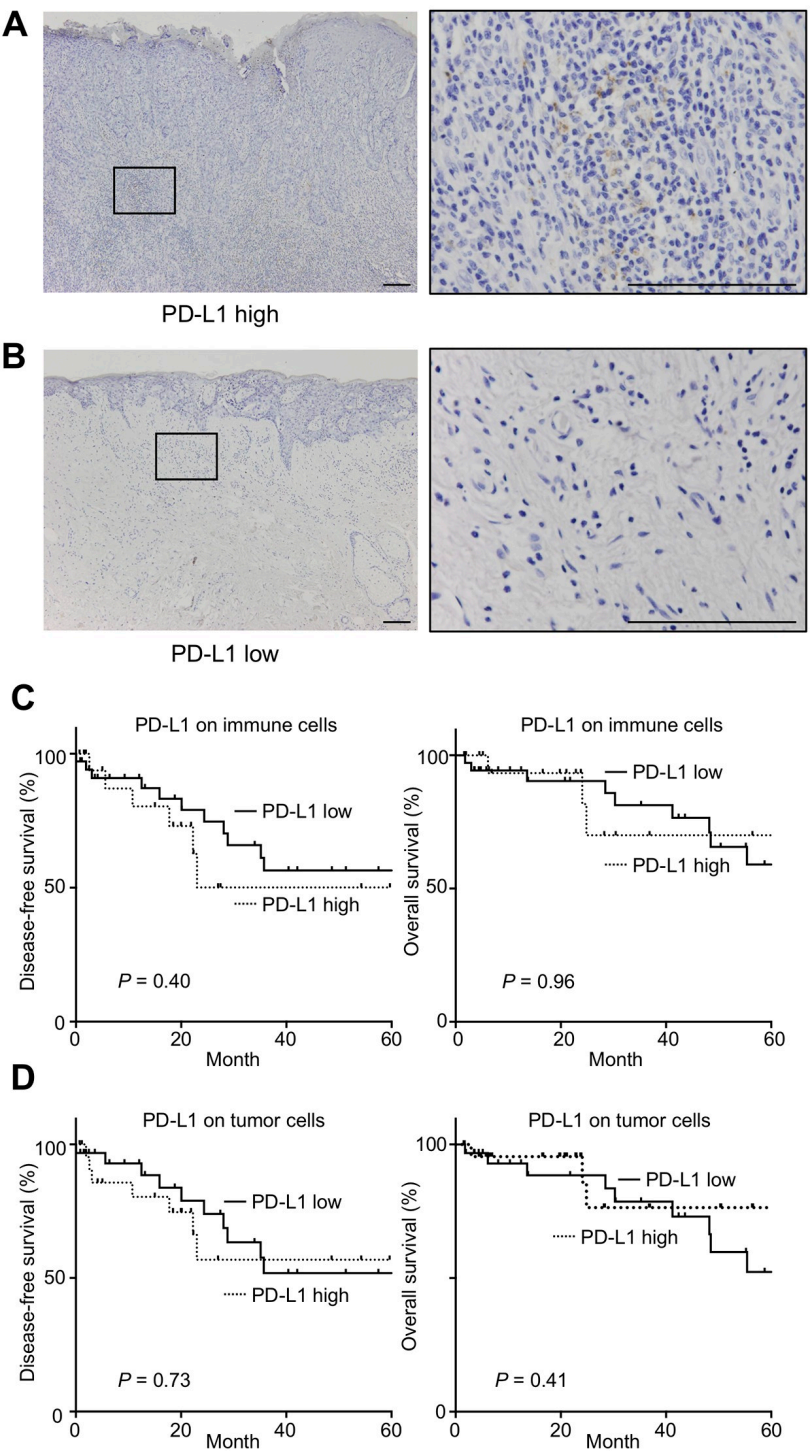
Kaplan-Meier curves of DFS and OS stratified by PD-L1 expression on immune cells and tumor cells. Representative IHC stainings of tumor specimens with high expression of PD-L1 on immune cells **(A)** and low expression of PD-L1 on immune cells **(B)** are shown. Scale bar, 100 μm. Kaplan-Meier DFS and OS curves stratified by PD-L1 expression on immune cells **(C)** and PD-L1 expression on tumor cells **(D)** are shown (n = 54). The log-rank test was performed for the univariate analysis.

Next, we assessed PD-L1 expression on tumor cells and immune cells in 54 EMPD patients by IHC (S1 Table). Immune cells and tumor cells were distinguishable on hematoxylin-eosin (HE) slides. Tumor cells were large with clear cytoplasm while immune cells were small with scant cytoplasm (S5 Fig). PD-L1+ tumor cells could be morphologically distinguished from PD-L1+ immune cells. PD-L1 was moderately expressed by immune cells, although PD-L1 expression by tumor was low (Fig 4A and 4B). PD-L1 expression in more than 5% of all immune cells was found in 19 cases (35.2%). PD-L1 expression in more than 5% of all tumor cells was found in only 1 case (1.85%), and PD-L1 expression in 1–5% of all tumor cells was found in 13 cases (24.1%). We examined the relationships between the PD-L1 expression on immune cells or tumor cells and prognosis (S1 Table). PD-L1 expression on tumor cells or immune cells was not associated with OS or DFS (Fig 4C and 4D). These results implicate that PD-1 and PD-L1 expressions might be irrelevant to the exhaustion of CD8+ TILs in EMPD.

### IDO was upregulated at the tumor site

**Fig 5:**
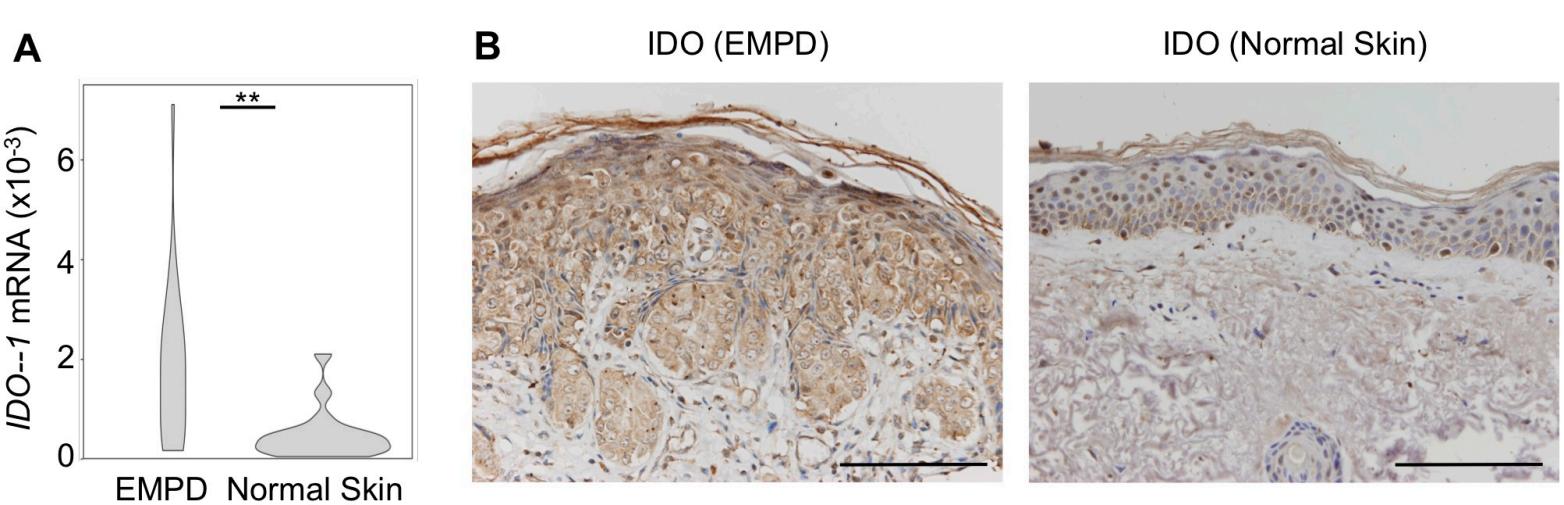
IDO expression at the tumor site. **(A)** mRNA expression of *IDO1* in tumor samples (n = 28) and normal skin samples (n = 18) using violin plots. **(B)** IHC of IDO in tumor samples and in normal skin samples. *P-*value was calculated by the Mann-Whitney U test between tumor samples and normal skin samples. Double asterisks indicate *P* < 0.01, scale bar, 100 μm.

PD-1/PD-L1 pathway is not the sole immunosuppressive pathway in cancer biology. There is mounting evidence that tumor cells produce IDO, which leads to the anergy of the effector CD8+ T cells [22]. We hypothesized that IDO expression contribute to T cell exhaustion in EMPD. Therefore, we next assessed whether EMPD tumor cells produced IDO. We compared the mRNA transcription level of *IDO1* in tumor samples from EMPD patients (S3 Table) and normal skin samples. Significantly higher mRNA expression of *IDO1* was observed in tumor samples than in normal skin samples (*P* < 0.01) (Fig 5A). Higher expression of IDO by morphologically atypical tumor cells and lower expression by keratinocytes from normal skin samples were observed by IHC (Fig 5B). These findings suggest that IDO expression by tumor cells might contribute to the exhaustion of CD8+ T cells in the tumor microenvironment of EMPD.

## Discussion

In this study, we found a decrease in the production of IFN-g, TNF-a, IL-2, perforin, and granzyme B in CD8+ TILs in EMPD, indicating that CD8+TILs in EMPD exhibited a functionally exhausted phenotype. Although PD-1 was upregulated on CD8+ TILs, the flow cytometric analysis showed that there was no correlation between the level of PD-1 expression on the CD8+ T cells and their functional ability. A high number of CD8+ cells was significantly associated with poor OS adjusted with age, sex, and clinical stage. On the other hand, the number of PD-1+ cells was not associated with either OS or DFS. Moreover, we found that tumor cells produced immunosuppressive molecule IDO.

In contrast to several previous reports [4,5], we found that a high number of CD8+ cells was associated with poor prognosis in EMPD. The paradoxical association between a high infiltration of CD8+ cells and poor prognosis was reported in renal cell carcinoma [23]. This report showed that a high infiltration of CD8+ cells and tumor grade were associated with poor prognosis and that there was a positive correlation between the number of CD8+ cells and tumor grade [23]. A high infiltration of CD8+ cells might predict the biological aggressiveness of EMPD, although there is no histologic grading system for EMPD to assess it.

It is commonly accepted that IDO suppresses the function of the effector CD8+T cells [22]. IDO contributes to the reduction of the level of cytotoxicity of CD8+ T cells [24]. On the other hand, several reports demonstrated that immune cells such as myeloid-derived suppressor cells (MDSCs) and Tregs contribute to CD8+ T cell dysfunction in the tumor microenvironment [25,26]. Therefore, these immune cells might affect CD8+ T cell dysfunction in EMPD as well. The interaction of these immune cells and CD8+ T cell dysfunction is of major interest in EMPD but we focused on the relationship between PD-1, PD-L1 and IDO-1 expression and T cell dysfunction in this study.

We consider that IDO expression at the tumor site is the primary mechanism by which T cell exhaustion is induced in EMPD patients. On the other hand, we found that the expression level of IFN-g in circulatory CD8+ T cells from EMPD patients was significantly lower than healthy controls. The effector functions of systemic CD8+ T cells might have partially lost effector functions in EMPD patients by the mechanisms we have not explored in this study. In addition, a previous report demonstrated that IDO-expressing plasmacytoid dendritic cells in tumor-draining lymph node induces the suppression of antigen-specific T cell response [27]. Therefore, in addition to IDO expression at the tumor site, there can be other immune-suppressive signals that impair effector functions of CD8+ T cells before they enter the tumor site.

When analyzing the cytotoxicity and effector functions of CD8+ T cells, we used peripheral blood and tumor tissue from the same patients in accordance with previous reports [6,7,15]. Our experimental design is limited in that we were unable to include uninvolved normal skin samples from the same patients for ethical reasons.

In summary, our work demonstrated that CD8+ TILs displayed an exhausted phenotype in EMPD. IDO expression seemed more relevant in inducing CD8 exhaustion than PD-1 upregulation or PD-L1 expression by immune cells. Restoring the effector functions of CD8+ TILs might be an effective treatment strategy for advanced EMPD.

## Supporting information

S1 Fig Perforin, granzyme B, IFN-g, TNF-a, and IL-2 expression in various CD8+ T cell subsets and types of samples (PBMCs or tumors). Freshly isolated PBMCs and digested tumor tissues from 10 EMPD patients were analyzed by flow cytometry. The effector functions with various T cells subsets and kinds of samples (PBMCs or tumors) were analyzed using a 2-way analysis of variance (ANOVA). *P-*values were calculated by the Wilcoxon signed-rank test between paired PBMCs and tumor tissues.
(PDF)

S2 Fig Perforin, granzyme B, IFN-g, TNF-a, and IL-2 expression in naïve CD8+ T cells of PBMCs from EMPD patients and healthy controls. Freshly isolated PBMCs from 10 EMPD patients and 7 healthy controls were analyzed by flow cytometry. Flow cytometric plots were pregated on TCRab+CD8+CD45RA+CCR7+ cells, excluding dead cells. The frequencies of the expression levels of perforin, granzyme B, IFN-g, TNF-a, and IL-2 in CD8+ T cells are shown. *P-*values were calculated by the Mann-Whitney U test between EMPD patients and healthy controls. A single asterisk indicates *P* < 0.05.
(PDF)

S3 Fig Perforin, granzyme B, IFN-g, TNF-a, and IL-2 expression in CD8+ T cells of PBMCs from EMPD patients and healthy controls. Freshly isolated PBMCs from 10 EMPD patients and 7 healthy controls were analyzed by flow cytometry. Flow cytometric plots were pregated on TCRab+CD8+ cells, excluding dead cells and naïve T cells (CD45RA+CCR7+ T cells). The frequencies of the expression levels of perforin, granzyme B, IFN-g, TNF-a, and IL-2 in CD8+ T cells are shown (n = 10). *P-*values were calculated by the Mann-Whitney U test between EMPD patients and healthy controls. Double asterisks indicate *P* < 0.01.
(PDF)

S4 Fig Representative flow cytometric plots of PD-1 expression among TCRab+ cells at the tumor site. Digested tumor tissues were analyzed by flow cytometry. Flow cytometric plots were pre-gated on TCRab+ cells, excluding dead cells. Representative flow cytometric plots of PD-1 expression among TCRab+ cells are shown.
(PDF)

S5 Fig Representative hematoxylin-eosin (HE) staining. Representative HE staining of tumor specimens is shown. Scale bar, 100 μm.
(PDF)

S1 Table Patient characteristics for survival analysis. (DOCX)

S2 Table Multivariate Cox-regression analysis including CD8 expression for overall survival. (DOCX)

S3 Table Patient characteristics of samples prepared for qPCR analysis. (DOCX)

S1 File Available data of survival analysis, qPCR, and flow cytometry. (XLSX)
