# Supplementary material for: Accumulation of exhausted CD8+ T cells in extramammary Paget’s disease
Source: PLoS One. 2019 Jan 25;14(1):e0211135. doi: 10.1371/journal.pone.0211135 (PMC6347258; doi:10.1371/journal.pone.0211135)
Supplement: S1 Fig — Freshly isolated PBMCs and digested tumor tissues from 10 EMPD patients were analyzed by flow cytometry. The effector functions with various T cells subsets and kinds of samples (PBMCs or tumors) were analyzed using a 2-way analysis of variance (ANOVA). P-values were calculated by the Wilcoxon signed-rank test between paired PBMCs and tumor tissues. (PDF) [file pone.0211135.s001.pdf]

Supplementary Figure 1

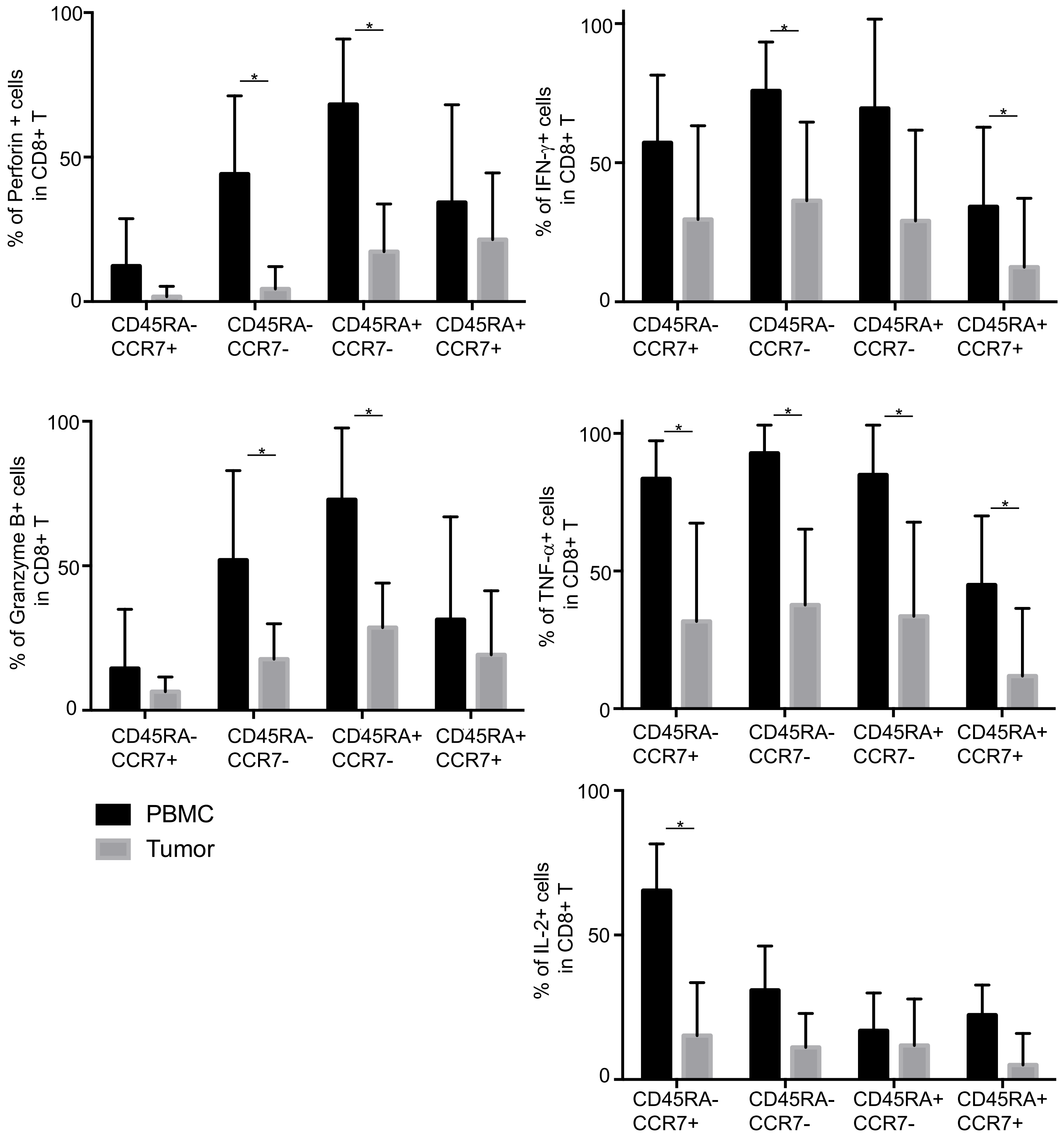

Perforin, granzyme B, IFN-γ, TNF-α, and IL-2 in various CD8+ T cell subsets and types of samples (PBMCs or tumors)
