# Supplementary material for: Accumulation of exhausted CD8+ T cells in extramammary Paget’s disease
Source: PLoS One. 2019 Jan 25;14(1):e0211135. doi: 10.1371/journal.pone.0211135 (PMC6347258; doi:10.1371/journal.pone.0211135)
Supplement: S2 Fig — Freshly isolated PBMCs from 10 EMPD patients and 7 healthy controls were analyzed by flow cytometry. Flow cytometric plots were pregated on TCRab+CD8+CD45RA+CCR7+ cells, excluding dead cells. The frequencies of the expression levels of perforin, granzyme B, IFN-g, TNF-a, and IL-2 in CD8+ T cells are shown. P-values were calculated by the Mann-Whitney U test between EMPD patients and healthy controls. A single asterisk indicates P < 0.05. (PDF) [file pone.0211135.s002.pdf]

Supplementary Figure 2

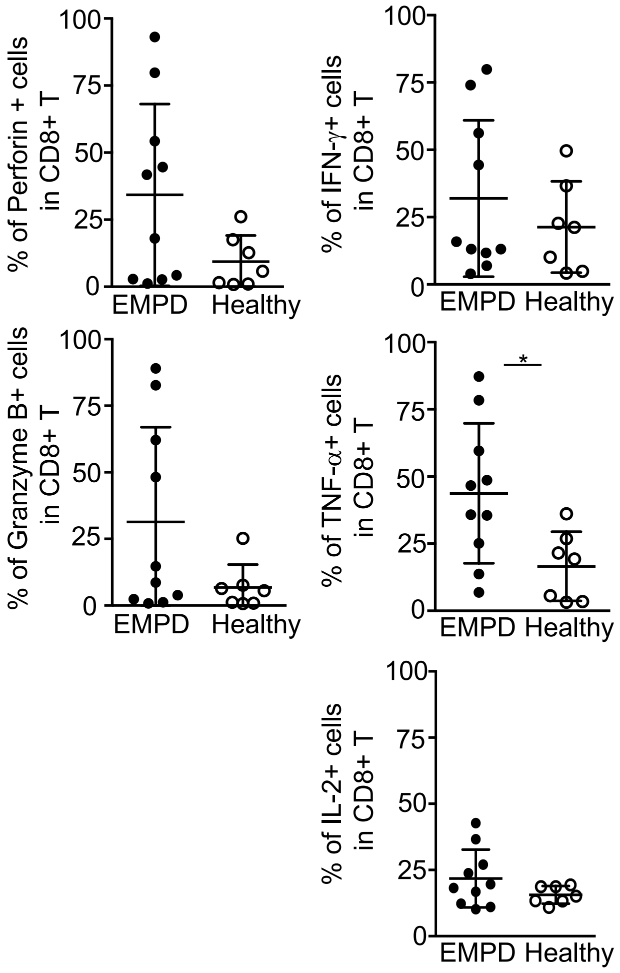

Perforin, granzyme B, IFN-γ, TNF-α, and IL-2 expression in naïve CD8+ T cells of PBMCs from EMPD patients and healthy controls
