# Supplementary material for: Accumulation of exhausted CD8+ T cells in extramammary Paget’s disease
Source: PLoS One. 2019 Jan 25;14(1):e0211135. doi: 10.1371/journal.pone.0211135 (PMC6347258; doi:10.1371/journal.pone.0211135)
Supplement: S3 Fig — Freshly isolated PBMCs from 10 EMPD patients and 7 healthy controls were analyzed by flow cytometry. Flow cytometric plots were pregated on TCRab+CD8+ cells, excluding dead cells and naïve T cells (CD45RA+CCR7+ T cells). The frequencies of the expression levels of perforin, granzyme B, IFN-g, TNF-a, and IL-2 in CD8+ T cells are shown (n = 10). P-values were calculated by the Mann-Whitney U test between EMPD patients and healthy controls. Double asterisks indicate P < 0.01. (PDF) [file pone.0211135.s003.pdf]

Supplementary Figure 3

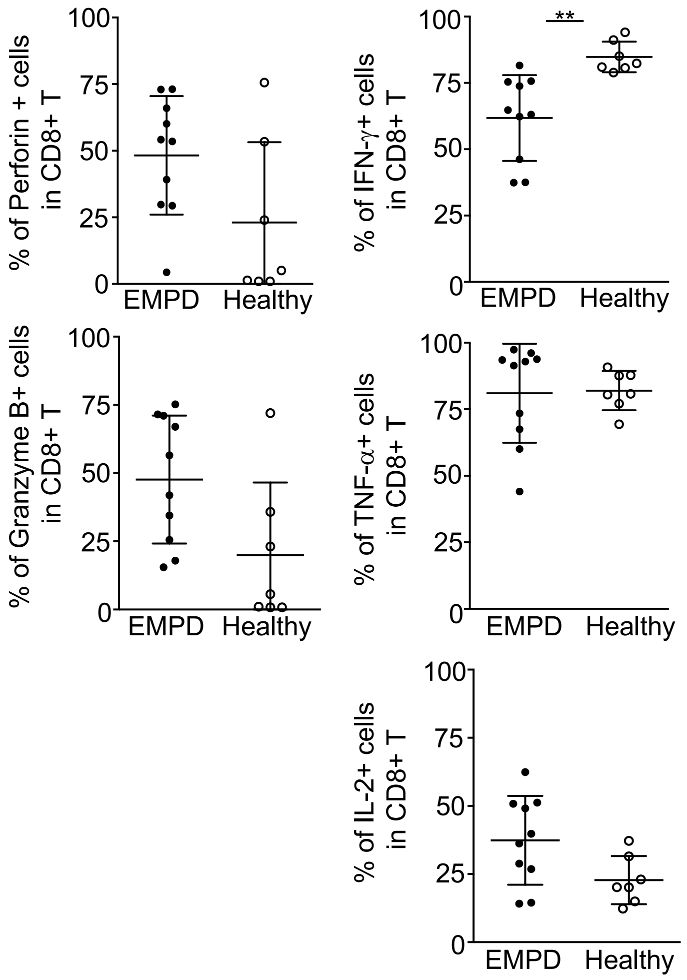

Perforin, granzyme B, IFN-γ, TNF-α, and IL-2 expression in CD8+ T cells of PBMCs from EMPD patients and healthy controls
