# Supplementary material for: Accumulation of exhausted CD8+ T cells in extramammary Paget’s disease
Source: PLoS One. 2019 Jan 25;14(1):e0211135. doi: 10.1371/journal.pone.0211135 (PMC6347258; doi:10.1371/journal.pone.0211135)
Supplement: S4 Fig — Digested tumor tissues were analyzed by flow cytometry. Flow cytometric plots were pre-gated on TCRab+ cells, excluding dead cells. Representative flow cytometric plots of PD-1 expression among TCRab+ cells are shown. (PDF) [file pone.0211135.s004.pdf]

Supplementary Figure 4

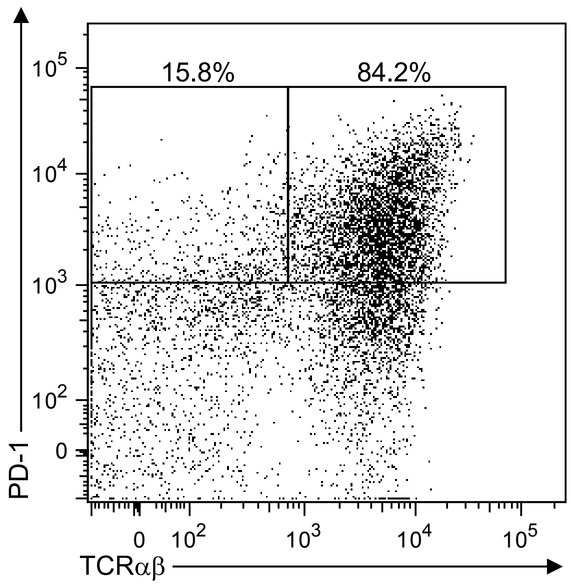

Representative flow cytometric plots of PD-1 expression among TCRαβ+ cells at the tumor site
