# Supplementary material for: Accumulation of exhausted CD8+ T cells in extramammary Paget’s disease
Source: PLoS One. 2019 Jan 25;14(1):e0211135. doi: 10.1371/journal.pone.0211135 (PMC6347258; doi:10.1371/journal.pone.0211135)
Supplement: S5 Fig — Representative HE staining of tumor specimens is shown. Scale bar, 100 μm. (PDF) [file pone.0211135.s005.pdf]

Supplementary Figure 5

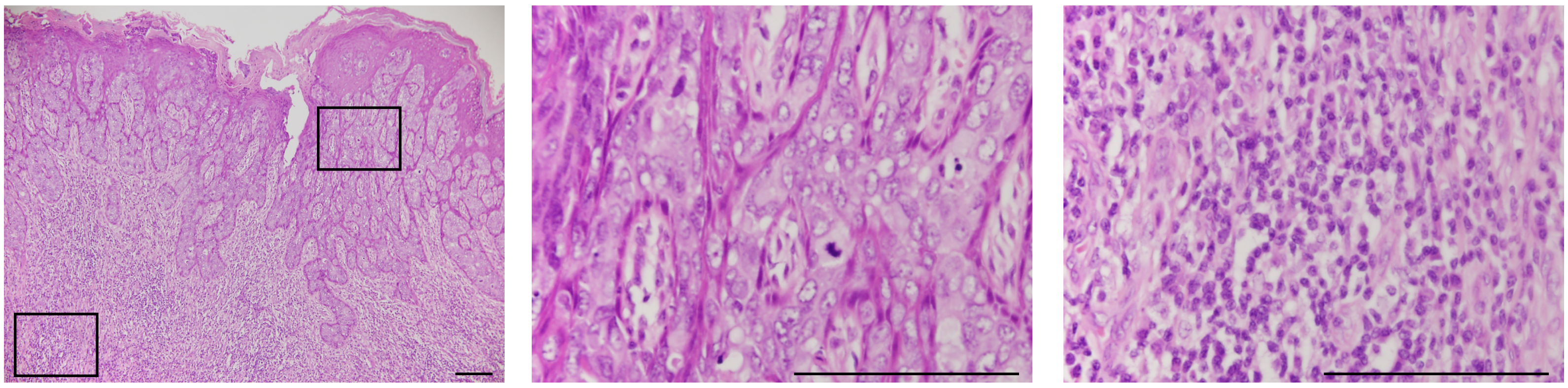

Tumor cells

Immune cells

Representative hematoxylin-eosin (HE) staining
