# Supplementary material for: Accumulation of exhausted CD8+ T cells in extramammary Paget’s disease
Source: PLoS One. 2019 Jan 25;14(1):e0211135. doi: 10.1371/journal.pone.0211135 (PMC6347258; doi:10.1371/journal.pone.0211135)
Supplement: S1 Table — (DOCX) [file pone.0211135.s006.docx]

**S1 Table. Patient characteristics for survival analysis**

| **Age** |  |  | **Stage** |  |
| --- | --- | --- | --- | --- |
| Median | 75 years |  | I | 21 |
| Range | 42-93 years |  | II | 11 |
| **Sex** |  |  | IIIa | 8 |
| Male | 44 |  | IIIb | 14 |
| Female | 10 |  |  |  |
| **Tumor Status** |  |  |  |  |
| T0 | 1 |  | **Lymphovascular Invasion** |  |
| T1 | 27 |  | present | 15 |
| T2 | 25 |  | **Follow-Up Period (Months)** |  |
| Unknown | 1 |  | Median | 24 months |
| **Nodal Status** |  |  |  |  |
| N0 | 32 |  | **Treatment** |  |
| N1 | 8 |  | Sentinel lymph node biopsy | 36 |
| N2 | 14 |  | Lymph node dissection | 19 |
|  |  |  | Chemotherapy (taxane) | 13 |
| **Primary Site** |  |  |  |  |
| Genitalia only | 47 |  |  |  |
| Genitalia and perianal | 4 |  |  |  |
| Perianal only | 2 |  |  |  |
| Axillary only | 1 |  |  |  |
