# Supplementary material for: Accumulation of exhausted CD8+ T cells in extramammary Paget’s disease
Source: PLoS One. 2019 Jan 25;14(1):e0211135. doi: 10.1371/journal.pone.0211135 (PMC6347258; doi:10.1371/journal.pone.0211135)
Supplement: S2 Table — (DOCX) [file pone.0211135.s007.docx]

**S2 Table. Multivariate Cox-regression analysis including CD8 expression for overall survival**

| Characteristics | HR | 95% CI | *P*-value |
| --- | --- | --- | --- |
| Age (/one year increase) | 1.03 | 0.95-1.12 | 0.41 |
| Female (vs. male) | 0.44 | 0.10-1.95 | 0.28 |
| Stage III (vs. I and II) | 2.51 | 0.75-8.30 | 0.13 |
| CD8 high (vs. CD8 low) | 5.03 | 1.03-24.4 | 0.045 |
