# Supplementary material for: Accumulation of exhausted CD8+ T cells in extramammary Paget’s disease
Source: PLoS One. 2019 Jan 25;14(1):e0211135. doi: 10.1371/journal.pone.0211135 (PMC6347258; doi:10.1371/journal.pone.0211135)
Supplement: S3 Table — (DOCX) [file pone.0211135.s008.docx]

**S3 Table. Patient characteristics of samples prepared for qPCR analysis**

| **Age** |  |  | **Stage** |  |
| --- | --- | --- | --- | --- |
| Median | 75 years |  | *in situ* | 9 |
| Range | 34-91 years |  | I | 8 |
| **Sex** |  |  | II | 6 |
| Male | 16 |  | IIIa | 0 |
| Female | 12 |  | IIIb | 3 |
|  |  |  | IV | 2 |
